# mTOR Pathway Inhibition, Anticancer Activity and In Silico Calculations of Novel Hydrazone Derivatives in Two- and Three-Dimensional Cultured Type 1 Endometrial Cancer Cells

**DOI:** 10.3390/ph17121562

**Published:** 2024-11-21

**Authors:** Muhammet Volkan Bulbul, Arif Mermer, Bircan Kolbasi, Fatih Kocabas, Semiha Mervenur Kalender, Kiymet Asli Kirectepe Aydin, Turan Demircan, İlknur Keskin

**Affiliations:** 1 Department of Histology and Embryology, School of Medicine, Agri Ibrahim Cecen University, Agri 04000, Turkey; mvbulbul@agri.edu.tr; 2 Department of Histology and Embryology, School of Medicine, Istanbul Medipol University, Istanbul 34810, Turkey; bkolbasi@medipol.edu.tr (B.K.); semiha.kalender@medipol.edu.tr (S.M.K.); 3 Department of Biotechnology, University of Health Sciences, Istanbul 34668, Turkey; 4 Experimental Medicine Application and Research Center, University of Health Sciences, Istanbul 34668, Turkey; 5 Department of Pharmacy, University of Health Sciences, Istanbul 34668, Turkey; 6 Department of Genetics and Bioengineering, Faculty of Engineering, Yeditepe University, Istanbul 34755, Turkey; faith.kocabas@yeditepe.edu.tr; 7 Department of Medical Biology and Genetics, Faculty of Medicine, Istanbul Nisantasi University, Istanbul 34398, Turkey; asli.kirectepeaydin@nisantasi.edu.tr; 8 Department of Medical Biology, School of Medicine, Mugla Sitki Kocman University, Mugla 48000, Turkey; turandemircan@mu.edu.tr

**Keywords:** mTOR, hydrazone, spheroid, endometrial cancer, molecular docking

## Abstract

Background: Endometrial cancer remains a significant health concern, with type 1 endometrial cancer characterized by aberrant expression of estrogen-dependent and mTOR pathway proteins. In this study, we evaluated the effects of two novel hydrazone derivatives against the Ishikawa cell line, a model for endometrial cancer. Methods: Two novel hydrazone derivatives, MVB1 and MVB2, were synthesized and characterized. The anticancer activity of the compounds in both two- and three-dimensional cultured Ishikawa cells was evaluated by MTT assay. The interaction of the compounds with proteins in the PI3K/AKT/mTOR pathway was evaluated by molecular docking studies and in vitro western blot analyses were performed. Additionally, ADME/T calculations were performed to evaluate the drug-like properties of the compounds. Results: MVB1 and MVB2 showed promising anticancer activity with IC_50_ values of 8.3 ± 0.5 µM and 9.0 ± 1.2 µM in 2D cultures, respectively, and 49.9 ± 2 µM and 20.6 ± 1.9 µM in 3D cultures, respectively. Molecular docking studies revealed significant interactions between these compounds and key proteins in the PI3K/AKT/mTOR pathway, with MVB1 exhibiting the highest mean binding score (−10.5 kcal/mol) among PI3K, AKT1, and mTOR proteins. In vitro studies confirmed that MVB1 effectively suppressed PI3K protein expression in both 2D and 3D cultures (*p* ≤ 0.0001). Conclusions: The findings suggest that MVB1 and MVB2, especially MVB1, are promising candidates for further development as potential therapeutics for endometrial cancer by targeting the PI3K/AKT/mTOR pathway.

## 1. Introduction

Approximately 380,000 new cases of endometrial cancer (EC) occur every year in the world and an average of 89,000 deaths are reported annually [1]. A 55% increase in incidence is predicted by 2030 [2]. Although the main treatment in EC cases is surgery, adjuvant radiotherapy and/or chemotherapy are used to reduce the risk of recurrence, depending on the stage of the cancer and other risk factors [3]. Multi-agent chemotherapy regimens such as paclitaxel/carboplatin, doxorubicin/cisplatin, paclitaxel/doxorubicin/cisplatin, docetaxel/carboplatin or bevacizumab/paclitaxel/carboplatin are used in the treatment of EC [4]. Apart from chemotherapeutics, mTOR inhibitors, which belong to the rapalog class, are accepted as a treatment option after chemotherapy due to the positive results they provide, although they are not yet universally widespread [5]. Among these, Rapamycin is the most widely accepted mTOR inhibitor. Rapamycin binds to the FK506 binding protein (FKBP12), forming a binary complex. This complex interacts with the FKBP12-rapamycin binding (FRB) domain of mTOR, causing structural changes that suppress the kinase activity of the mTORC1 complex [6].

Ishikawa cells have been used as a model for Type I EC since their identification [7]. The PI3K-AKT-mTOR pathway is the most frequently abnormal pathway in type I tumors, as 30% to 80% of sporadic endometrioid ECs harbor a PTEN mutation [8,9,10]. Activation of the PI3K-AKT-mTOR pathway occurs through loss of PTEN tumor suppressor function [11,12]. Additionally, increased activation of the PI3K-AKT-mTOR pathway is observed in the presence of AKT and PIK3CA mutations, which are common in type I tumors [13]. Studies on the discovery of new chemicals targeting PI3K-AKT-mTOR pathway proteins in EC treatment are becoming the focus of attention [14].

**Figure 1:**
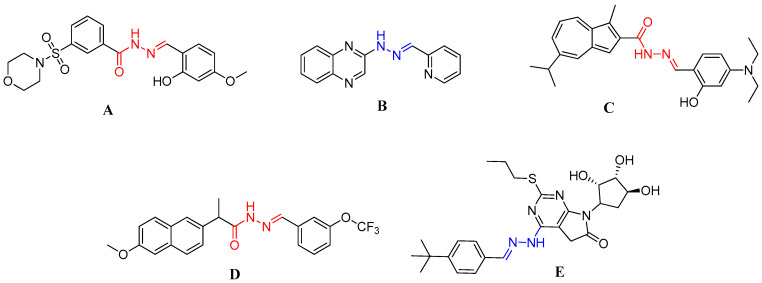
Reported hydrazone derivatives as anticancer agents against different cell lines.

The acylhydrazone scaffold (-CONHNH=) has garnered significant attention for decades owing to its diverse applications, ranging from pharmaceuticals and agrochemicals to functional materials [15,16]. Recently, numerous compounds containing this moiety have been reported, indicating that incorporation of this pharmacophore can lead to highly promising activities such as antiviral, antibacterial, antitumor, anticancer, antioxidant and antileishmanial effects [17,18,19,20,21]. Accordingly, several studies have highlighted the crucial role of the hydrazone moiety in the development of anticancer drug candidates. In a recent study conducted by Li and co-workers, sulfonamide-based hydrazone derivatives were synthesized, and Compound A was reported to exhibit strong anti-proliferative effects on EC9706 and EC109 esophageal carcinoma cells, with IC_50_ values of 1.09 ± 0.03 and 2.79 ± 0.45 μM, respectively [22]. In another study, in which quinoxalinyl-hydrazone derivatives were synthesized and different cancer cell lines investigated, compound B showed antiproliferative activity against HCT116 cells suppressing cell growth in the time- and dose-dependent manner (Figure 1) [23].

The majority of drug candidate trials are conducted on cultured cells grown in two dimensions (2D) on plastic surfaces. However, convincing evidence indicates that cells cultured under these non-physiological conditions do not accurately represent those found within the complex microenvironment of tissues [24]. As a result, three-dimensional (3D) cell culture techniques, which more closely mimic the in vivo cellular environments, have gained attention because they are anticipated to offer improved sensitivity in drug discovery [25].

In this study, two different compounds (MVB1 and MVB2) containing acylhydrazone scaffolds, determined by the molecular docking method (for PI3K, Akt, and mTOR proteins), were synthesized. Following in silico ADME (absorption, distribution, metabolism, excretion) and toxicity analyses, the anticancer effects of the compounds on 2D and 3D cultured Ishikawa cells and the changes in PI3K, Akt, and mTOR protein levels were examined in vitro in comparison with rapamycin.

## 2. Results

### 2.1. Chemistry

**Figure 2:**
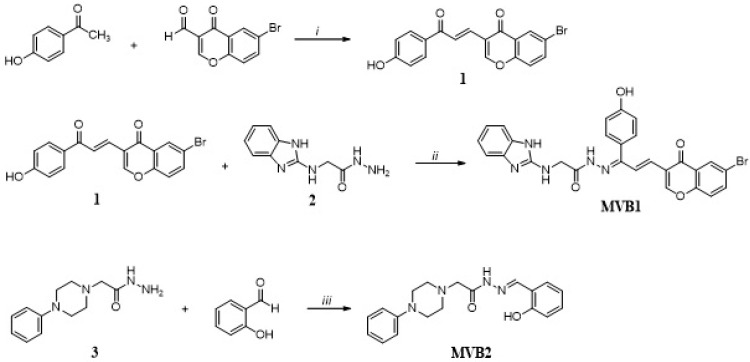
Synthesis of target compounds; (i) NaOH, EtOH, (ii) AcOH, (iii) EtOH, AcOH.

For the synthesis of MVB1, the first of the target compounds, compound (**1**) was obtained by aldol condensation between 6-bromo-4-oxo-4H-chromene-3-carbaldehyde and 4-hydroxy acetophenone. Then, the previously synthesized hydrazide compound (**2**) [26] was reacted with the obtained chalcone derivative in the presence of glacial acetic acid. In order to obtain the second target compound, 2-(4-phenylpiperazin-1-yl)acetohydrazide (**3**) synthesized by our group [27] was treated with 2-hydroxy benzaldehyde. The compound MVB2 is a Schiff base derivative and was achieved by imine bond formation (-N=CH). Both target compounds were synthesized with excellent yields (Figure 2).

FTIR spectra of the synthesized compounds MVB1 and MVB2, the disappearance of the -NH2 vibration bands in the hydrazide compounds and the appearance of -NH bands, the presence of bands belonging to the carbonyl group, and also the appearance of the (-N=CH) bands arising from the imine bond as a new band in the range 1612–1633 cm^−1^ supported the structures of the compounds [28]. The imine proton was resonated at 8.05 ppm for MVB1 and 8.68 ppm for MVB2 in 1H NMR spectra. The corresponding carbon peaks were observed at 156.99 ppm and 148.30 ppm in 13C NMR spectra, respectively. In addition, the disappearance of amine protons (-NH2) and the observation of NH protons confirmed the molecular structure of the compounds. These protons were resonated at 11.23 ppm and 11.02 ppm for MVB1 and MVB2, respectively. Considering MS results of the compounds, the obtained data supported the formation of target compounds with [M + 1]^+^ and/or [M + Na]^+^ ion peaks. All characterization results were given in (Figure S1).

### 2.2. Exploring Binding Affinities and Molecular Interactions of mTOR Pathway Inhibitors Through Docking Studies

**Figure 3:**
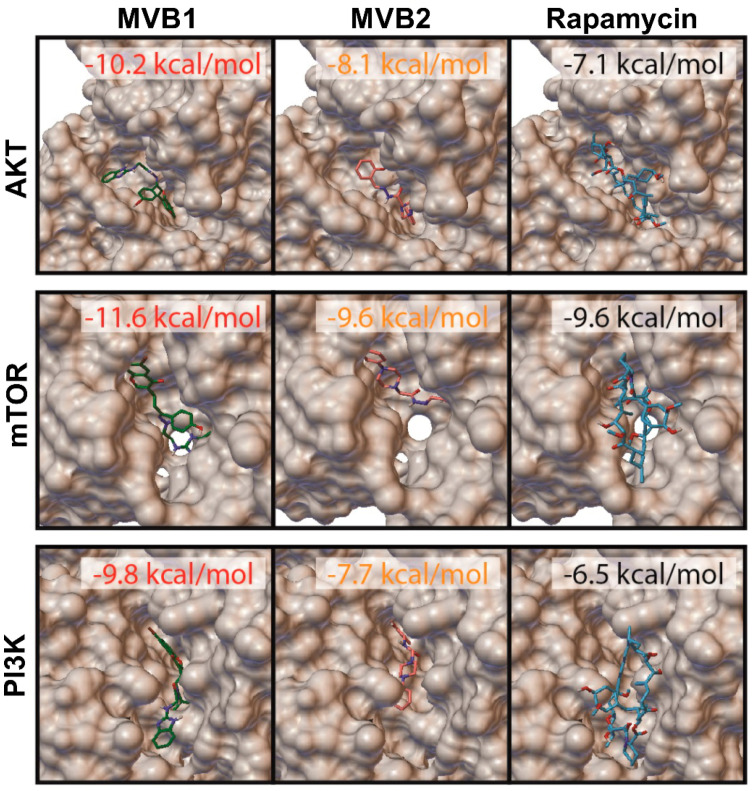
Three-dimensional target and ligand pose for MVB1, MVB2, and Rapamycin.

The binding affinities of the compounds to the relevant proteins were calculated. The inhibition potentials of the compounds Rapamycin [29], Copanlisib [30], Cromolyn [31], GSK690693 [32], Myricetin [33], Naproxen [34], Perifosine [35], PF-04802367 [36], Quercetin [37], and Wortmannin [38] are known to act on various proteins in the mTOR pathway. Among the compounds tested in this study, MVB1 demonstrated the highest binding affinity, surpassing rapamycin and all other known mTOR pathway inhibitors. MVB1 exhibited values of −9.8 kcal/mol for PI3K, −10.2 kcal/mol for AKT, and −11.6 kcal/mol for mTOR. MVB2 displayed moderate binding affinities, with values of −7.7 kcal/mol for PI3K, −8.1 kcal/mol for AKT, and −9.6 kcal/mol for mTOR. Rapamycin exhibited relatively weaker binding compared to MVB1 as well as MVB2 but displayed higher affinities than some of the other known inhibitors (Table 1). This structural analysis plays a pivotal role in elucidating the mechanism of action of the compounds and assessing their potential as inhibitors of mTOR signaling. To this end, three-dimensional target and ligand poses were examined between the compounds MVB1, MVB2, and rapamycin with their respective targets within the mTOR pathway (Figure 3). These compounds exhibited binding affinities that indicate they fit well into the catalytic domains of the proteins involved.

### 2.3. ADME and Toxicity Calculation

ADME analysis involves examining the absorption and distribution of molecules within human metabolism, their interactions and effects during metabolic processes, and their excretion from the body [39]. The analysis yielded numerous parameters. The calculated ADME parameters for the compounds MVB1 (Figure S2), MVB2 (Figure S3), and rapamycin (Figure S4) are provided as Supplementary Material. However, as each parameter evaluates a different property, analyzing the compounds based solely on individual parameters may prove challenging.

All these parameters are evaluated in the SwissADME program according to the criteria suggested by Lipinski, Ghoose, Veber, Egan and Muegge, and their suitability for each is revealed. Accordingly, the bioavailability score is calculated. In this scoring, if the compound has a value greater than zero, it is considered physiologically active. Additionally, based on these results, a bioavailability radar was created by the SwissADME (2023) program. The colored area in the middle of the radar indicates the physicochemical area suitable for oral bioavailability. The more a compound can be included within the boundaries of this field, the higher the drug similarity [40].

**Figure 4:**
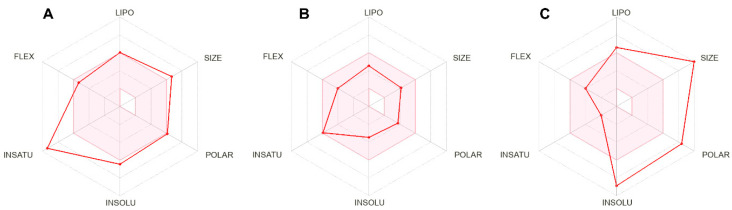
Images of bioavailability radars. (**A**) MVB1, (**B**) MVB2, (**C**) Rapamycin. (Lıpo: lipophilicity, Polar: polarity, Insolu: insolubility, Flex: flexibility, Insatu: insaturation).

The bioavailability score was determined as 0.55 for MVB1 and MVB2, and 0.17 for rapamycin. Based on these results, it can be suggested that both compounds are physiologically active and have greater bioavailability potential than rapamycin. Although MVB1 and MVB2 compounds have the same bioavailability score, MVB2 was more successful in being included within the bioavailability radar limits (Figure 4). Compared to rapamycin, the bioavailability radars of the synthesized target compounds are more compliant with the determined criteria, both because the molecules have a smaller molecular weight, higher solubility and better flexibility.

The web-based ProTox-3.0 program was used for toxicity assessment. Toxicity prediction results for MVB1 (Figure S5), MVB2 (Figure S6) and Rapamycin (Figure S7) are given as Supplementary Material. ProTox-3.0 provides toxicity evaluation in 45 different parameters. By analyzing data across multiple parameters simultaneously, the results page displays the estimated median lethal dose (LD50) in mg/kg body weight, the toxicity class, and the prediction accuracy. While MVB1 and rapamycin showed class 5 toxicity, MVB2 was included in class 4. Accordingly, it can be said that the MVB1 compound will be a more successful candidate in future in vivo studies. When evaluated in terms of prediction accuracies and average similarity, rapamycin exhibited a 100% accuracy level, while much lower rates were detected in MVB1 and MVB2 compounds (Table 2).

### 2.4. In Vitro Cytotoxicity in 2D and 3D Cell Culture

IC_50_, LogIC_50_ values exhibited by 2D and 3D cultured Ishikawa cells exposed to various doses of MVB1, MVB2 and in for 24 h are shown in Table 3. Compounds MVB1 (IC_50_: 8.3/±0.5 µM) and MVB2 (IC_50_: 9.0/±1.2 µM) exhibited cytotoxicity potential in 2D cultured Ishikawa cells. However, rapamycin showed significantly lower cytotoxicity potential than MVB1 and MVB2 compounds with its IC_50_ value of 21.5/±0.6. The IC_50_ values of MVB1, MVB2 and rapamycin in 3D cultures (49.9/±2.8 µM, 20.6/±1.9 µM, 38.5/±2.0 µM, respectively) were clearly higher than in 2D cultures.

**Figure 5:**
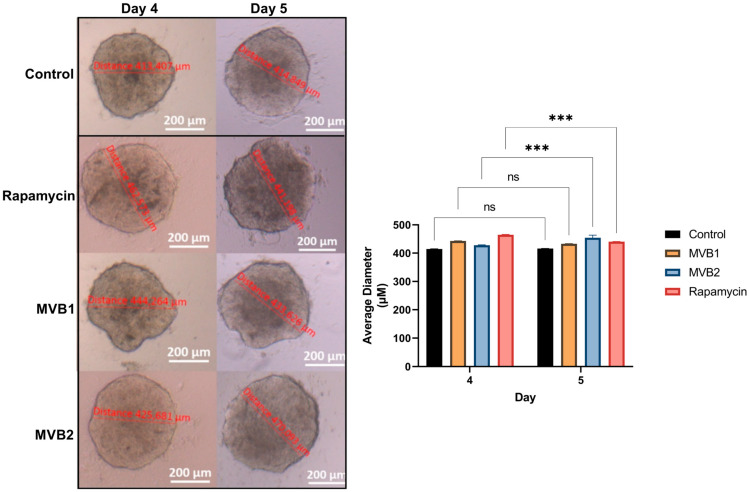
Statistical graphs of spheroid images and average diameter changes before and after exposure to the IC_50_ values of the compounds and rapamycin. (***: *p* ≤ 0.001, ns: non significant). (Magnification: 4×).

The average diameter measurements of spheroids exposed to IC_50_ values before (Day 4) and after (Day 5) were investigated (Figure 5). Although the spheroid diameter increased from 413.407 µm to 414.849 µm in the control group, no statistically significant difference was observed in the average diameter (ns: *p* > 0.05). Similarly, although the diameter decreased from 444.264 µm to 433.626 µm in the MVB1 group, no statistically significant difference was observed in the average diameter. In the rapamycin group, the mean diameter decreased significantly after exposure (***: *p* ≤ 0.001). Contrary to expectations, the mean diameter in the MVB2 group increased statistically significantly after exposure (***: *p* ≤ 0.001).

**Figure 6:**
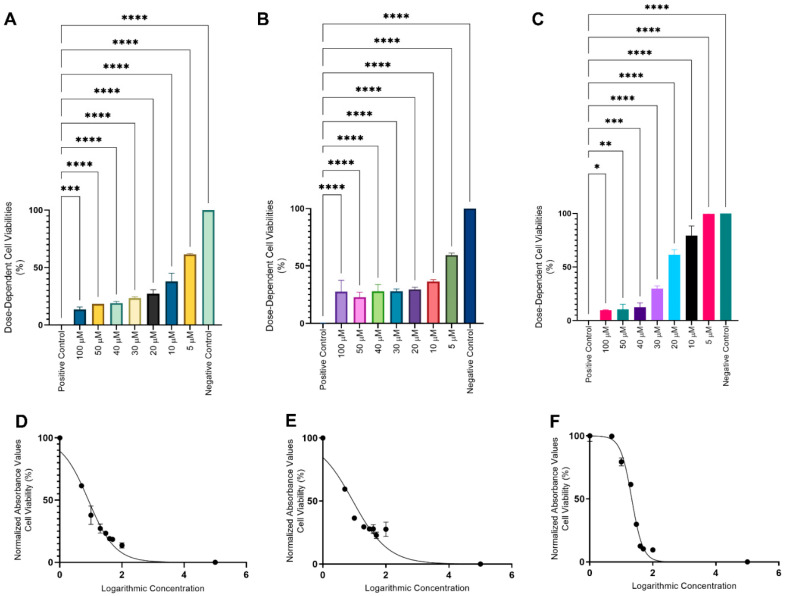
Dose-dependent differences in cell viability and viability curves of MVB1, MVB2, and rapamycin in 2D cultures. (**A**,**D**) MVB1, (**B**,**E**) MVB2, (**C**,**F**) Rapamycin. (****: *p* ≤ 0.0001, ***: *p* ≤ 0.001, **: *p* ≤ 0.01, *: *p* ≤ 0.05).

The effects of MVB1, MVB2 and rapamycin on viability percentages according to doses in 2D cultures and viability curve graphs are given in Table 4 and Figure 6. There is usually a well-defined relationship between drug dose and drug effect, expressed by a dose-response curve [41]. When evaluated in this context, MVB1 and rapamycin caused a decrease in cell viability in accordance with the increasing dose. However, MVB2 failed to demonstrate the same potential. While it showed a consistent decrease in the percentage of viability from the 5 µM dose to the 50 µM dose, the 30 µM and 40 µM doses caused the same effect. Additionally, contrary to expectations, a higher viability percentage was recorded at the 100 µM dose than at the 50 µM dose (Table 4) (Figure 6B,E).

**Figure 7:**
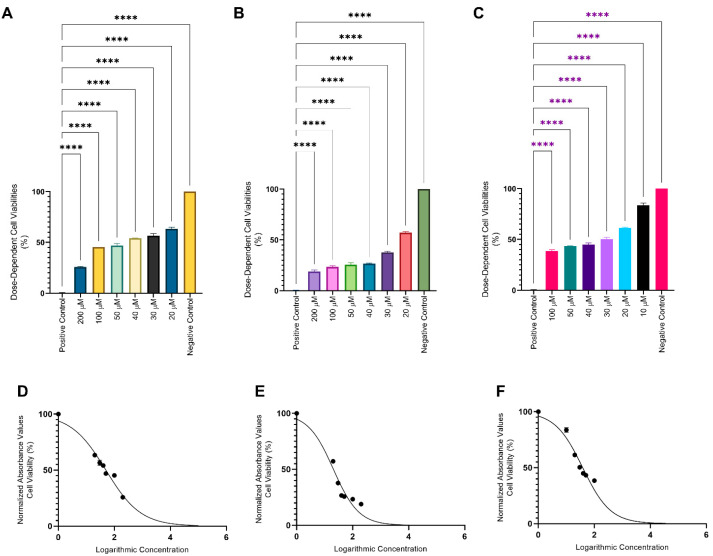
Dose-dependent differences in cell viability and viability curves of MVB1, MVB2, and rapamycin in 3D cultures. (**A**,**D**) MVB1, (**B**,**E**) MVB2, (**C**,**F**) Rapamycin. (****: *p* ≤ 0.0001).

The effects of MVB1, MVB2 and rapamycin on viability percentages according to doses in 3D cultures and viability curve graphs are given in Table 5 and Figure 7. The compounds and rapamycin successfully caused a decrease in viability with increasing dose. Since 3D cultures are known to have high tolerance to chemicals, dose ranges were studied differently than 2D cultures (20–200 µM and 10–100 µM). Minimum and maximum doses have been increased. Due to the disintegration of the spheroid structure in 3D cultured Ishikawa cells exposed to a 200 µM dose of rapamycin, this dose was not included in the analyses.

### 2.5. PI3K, Akt, and mTOR Protein Levels in 2D and 3D Cultures

**Figure 8:**
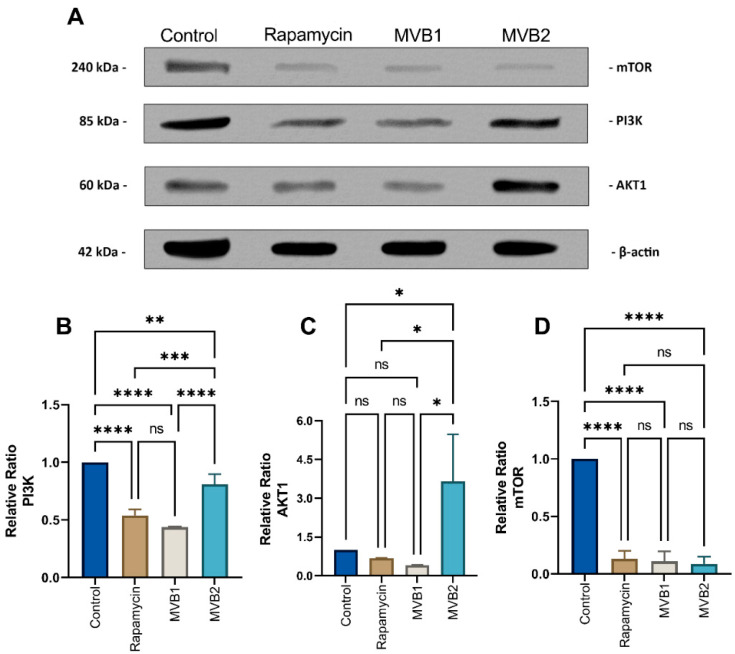
Representative Western blot analysis of PI3K, AKT1, and mTOR expression in 2D cultured cells. (**A**) Representative blots are also shown. Relative ratios of (**B**) PI3K, (**C**) AKT1, and (**D**) mTOR versus beta-actin were measured and normalized. Data are presented as mean ± standard error of the mean (three independent spots per group). Beta-actin was used as a loading control. (****: *p* ≤ 0.0001, ***: *p* ≤ 0.001, **: *p* ≤ 0.01, *: *p* ≤ 0.05, ns (non significant): *p* > 0.05).

In the control group, an increase in PI3K levels was observed in 2D cultures compared to the groups treated with MVB1 (*p* ≤ 0.0001), MVB2 (**: *p* ≤ 0.01), and rapamycin (*p* ≤ 0.0001). No significant difference in PI3K levels was found between the rapamycin and MVB1 groups (ns: *p* > 0.05). The PI3K levels in cells treated with the MVB2 compound were significantly higher than those in the MVB1 (**: *p* ≤ 0.0001) and rapamycin (*p* ≤ 0.001) groups (Figure 8B). When examining AKT1, no significant differences were detected among the control, MVB1, and rapamycin groups (ns: *p* > 0.05). However, the AKT1 protein levels in cells treated with the MVB2 compound were significantly elevated compared to all other groups (*p* ≤ 0.05) (Figure 8C). For mTOR, there were no significant differences between the rapamycin, MVB1, and MVB2 groups (ns: *p* > 0.05), but the mTOR levels in the control group were significantly higher than those in the other groups (****: *p* ≤ 0.0001) (Figure 8D).

**Figure 9:**
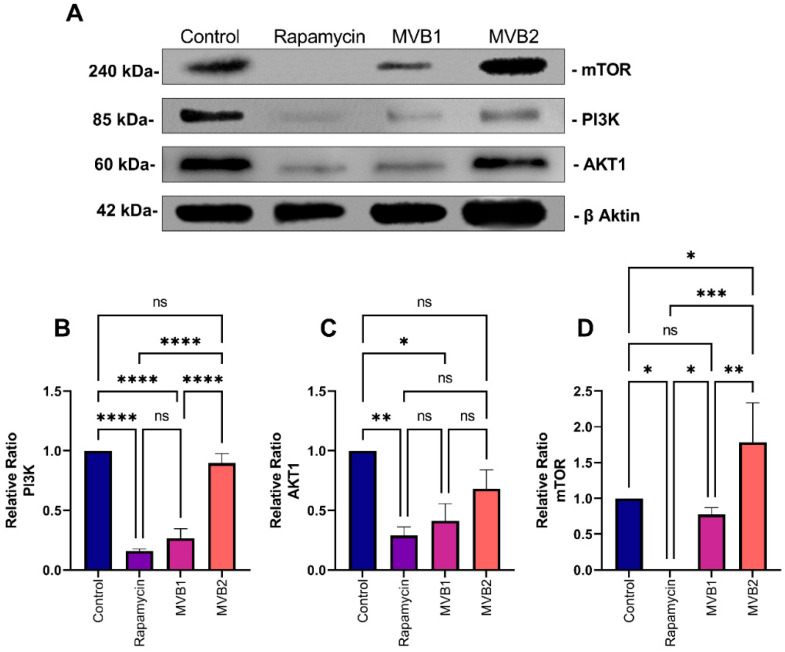
Representative Western blot analysis of PI3K, AKT1, and mTOR expression in 3D cultured cells. (**A**) Representative blots are also shown. Relative ratios of (**B**) PI3K, (**C**) AKT1, and (**D**) mTOR versus beta-actin were measured and normalized. Data are presented as mean ± standard error of the mean (three independent spots per group). Beta-actin was used as a loading control (****: *p* ≤ 0.0001, ***: *p* ≤ 0.001, **: *p* ≤ 0.01, *: *p* ≤ 0.05, ns (non significant): *p* > 0.05).

In 3D cultured cells, PI3K levels were elevated in the control group when compared to the MVB1 and rapamycin groups ( *p* ≤ 0.0001). No significant differences were observed between the rapamycin and MVB1 groups (ns: *p* > 0.05), nor between the control group and MVB2 (ns: *p* > 0.05) (Figure 9B). For AKT1 levels, no significant differences were noted among the MVB1, MVB2, and rapamycin groups (ns: *p* > 0.05). However, when comparing rapamycin (**: *p* ≤ 0.01) and MVB1 (*p* ≤ 0.05) to the control group, significantly higher AKT1 levels were found in the control group. No significant difference was identified between the control and MVB2 groups (ns: *p* > 0.05) (Figure 9C). Regarding mTOR levels, no significant differences were seen between the control and MVB1 groups (ns: *p* > 0.05). A significant increase was noted in the control group compared to rapamycin (*p* ≤ 0.05), as well as a significant increase in the MVB2 group when compared to the control (*p* ≤ 0.05). The MVB2 group exhibited significantly higher mTOR levels than the other groups, specifically compared to rapamycin (*p* ≤ 0.001) and MVB1 (**: *p* ≤ 0.01) (Figure 9D).

### 2.6. PI3K, Akt, and mTOR Proteins Fluorescence Intensities in 2D and 3D Cultures

**Figure 10:**
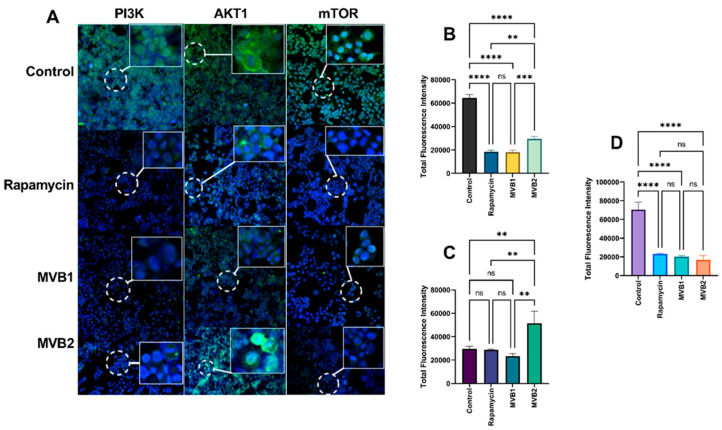
(**A**) Representative immunofluorescence staining analysis of PI3K, AKT1, and mTOR expression in 2D cultured cells. Total fluorescence intensity ratios of (**B**) PI3K, (**C**) AKT1, and (**D**) mTOR. Data are presented as mean ± standard error of the mean (****: *p* ≤ 0.0001, ***: *p* ≤ 0.001, **: *p* ≤ 0.01, ns (non significant): *p* > 0.05).

Immunofluorescence staining was conducted to semi-quantitatively validate the results obtained from Western blot analyses. Measurements were taken from 10 randomly selected areas in the fluorescence images of 2D cultures and utilized for statistical analysis. The fluorescence expression level of PI3K in 2D cultured cells was significantly higher in the control group compared to the rapamycin, MVB1, and MVB2 groups (*p* ≤ 0.001). The MVB2 group demonstrated the second highest PI3K expression after the control group. No significant difference was found between the rapamycin and MVB1 groups (ns: *p* > 0.05) (Figure 10B). The AKT1 level was significantly elevated in the MVB2 group compared to the rapamycin, MVB1, and control groups (*p* ≤ 0.01). However, there was no significant difference among the rapamycin, MVB1, and control groups (ns: *p* > 0.05) (Figure 10C). The mTOR level was found to be significantly higher in the control group than in the rapamycin, MVB1, and MVB2 groups (***: *p* ≤ 0.0001). No significant differences were observed between the rapamycin, MVB1, and MVB2 groups (ns: *p* > 0.05) (Figure 10D). All of these findings were consistent with the Western blot results in 2D cultures.

**Figure 11:**
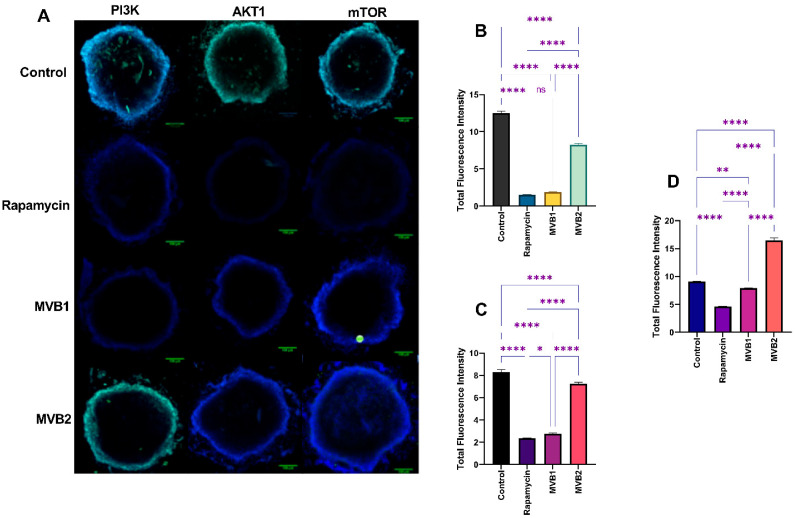
(**A**) Representative immunofluorescence staining analysis of PI3K, AKT1, and mTOR expression in 3D cultured cells. Total fluorescence intensity ratios of (**B**) PI3K, (**C**) AKT1, and (**D**) mTOR. Data are presented as mean ± standard error of the mean (****: *p* ≤ 0.0001, **: *p* ≤ 0.01, *: *p* ≤ 0.05, ns (non significant): *p* > 0.05).

In 3D cultures, the fluorescence expression level of PI3K was found to be higher in the control group compared to the rapamycin, MVB1, and MVB2 groups (*p* ≤ 0.0001). The MVB2 group showed the second highest PI3K levels after the control group, with no significant difference observed between the rapamycin and MVB1 groups (ns: *p* > 0.05) (Figure 11B). The AKT1 level was also higher in the control group than in the rapamycin, MVB1, and MVB2 groups (*p* ≤ 0.0001), with MVB2 showing the highest AKT1 level after the control group. A lower expression of AKT1 was noted in the rapamycin group compared to the MVB1 group (*p* ≤ 0.05) (Figure 11C). The mTOR level was significantly higher in the MVB2 group than in the rapamycin, MVB1, and control groups (*p* ≤ 0.0001), with the control group exhibiting the highest mTOR level after the MVB2 group. A significantly lower mTOR level was recorded in the rapamycin group compared to the MVB1 group (*p* ≤ 0.001) (Figure 11D). All of these results were in line with the Western blot findings in 3D cultures.

## 3. Discussion

Type 1 EC are estrogen dependent, and estrogen induces the mTOR signaling pathway, which contributes to the proliferation of EC cells [42]. Therefore, the compounds synthesized in our study were investigated against Ishikawa cells representing Type 1 EC. In cytotoxicity models derived from the Chemical European Biological Laboratory (ChEMBL) database, any compound with an IC50 value of 10 µM or lower in an in vitro toxicity assay against HepG2 cells (a human liver cancer cell line) is classified as positively cytotoxic [43,44]. Similarly, compounds MVB1 (IC_50_: 8.3/±0.5 µM) and MVB2 (IC_50_: 9.0/±1.2 µM) exhibited cytotoxicity potential in 2D cultured Ishikawa cells. However, rapamycin showed significantly lower cytotoxicity potential than MVB1 and MVB2 compounds with its IC_50_ value of 21.5/±0.6. The overall evaluation of rapamycin as a cytostatic rather than a cytotoxic compound [45] may explain this result.

Carboplatin is one of the leading agents used in conventional adjuvant EC treatment. In a prior study, the IC50 value of carboplatin against the 2D-cultured Ishikawa cell line was found to be 500 µM [46], which is significantly higher than the IC50 values of MVB1 and MVB2, the compounds investigated in our study. The differences in the physical and physiological characteristics between 2D and 3D cultures result in 3D cells exhibiting greater drug resistance [47]. This is because, in 2D cultured cells, various complex biological functions such as invasion, apoptosis, transcriptional regulation, receptor expression [48,49], cell proliferation and anti-apoptosis [50,51] are forced to be altered. The large differences recorded between IC_50_ values in 2D and 3D cultures in our study prove this.

To improve outcomes in women with type I EC, it may be more beneficial to combine treatment with endocrine therapy, potentially blocking a pathway known as the PI3K/AKT/mTOR pathway [52]. In this way, problems related to side effects and toxicities of chemotherapeutics can be avoided as much as possible. Therefore, in our study, the effects of MVB1 and MVB2 compounds on mTOR pathway proteins in Ishikawa cells representing Type 1 EC were evaluated in comparison with rapamycin. It is known that there are gene expression differences as well as drug sensitivities between 3D culture systems and 2D culture systems [53]. A study involving prostate cancer cell lines discovered that compounds aimed at the mTOR pathway were able to inhibit cancer cells in both 2D and 3D cultures, whereas those targeting the AKT pathway showed reduced effectiveness in 2D cultures [54]. These changes in 2D and 3D models were also revealed in our study. Namely, while MVB1 and MVB2 compounds exhibited dual mTOR/PI3K inhibitor potential according to 2D western blot results, the situation was different in 3D models. In 3D cultures, the MVB1 compound failed to suppress mTOR compared to rapamycin and the control group. However, AKT1 reduced protein expressions and maintained its effect on PI3K. However, MVB2 could not cause changes in PIK3, AKT1 and mTOR levels in 3D. In a study comparing 2D and 3D cultures of colon cancer cells, it was shown that PI3K/mTOR signaling was at lower levels in 3D cultures compared to 2D cultures [55]. The fact that rapamycin was more successful in inhibiting mTOR in 3D cultures compared to 2D cultures in our study may be related to this.

## 4. Materials and Methods

### 4.1. Materials and Instrumentation

All chemicals were acquired from Sigma Aldrich and were used as received without any additional purification. Reactions were monitored using Thin Layer Chromatography (TLC) on silica gel 60 F254 aluminum plates. FTIR (Fourier Transform Infrared Spectroscopy) spectra were recorded with a ThermoFisher Scientific Nicolet IS50 FTIR spectrometer (Catalog number 912A0760). Spectra 1H NMR and 13C NMR were obtained in DMSO-d6 using a BRUKER AVANCE II 400 MHz NMR spectrometer (operating at 400.13 MHz for 1H and 100.62 MHz for 13C). Mass spectra were collected using MALDI-TOF/MS (UltrafleXtreme, Bruker, Billerica, MA, USA).

### 4.2. Synthesis

#### 4.2.1. 2-[(1H-1,3-Benzodiazol-2-yl)amino]-N′-[(1Z,2E)-3-(6-Bromo-4-oxo-4H-Chromen-3-yl)-1-(4-Hydroxyphenyl)prop-2-en-1-Ylidene]acetohydrazide (MVB1)

To a solution of 6-bromo-4-oxo-4H-chromene-3-carbaldehyde (10 mmol) in ethanol was added 4-hydroxyacetophenone (10 mmol) and NaOH (50%, 3 mL) in ethanol. The reaction mixture was stirred at room temperature for 24 h. After completion of the reaction, the mixture was poured into ice water and neutralized with 1N HCl (aq). The resulting yellow solid was filtered, and washed slightly with water. The crude solid was purified with recrystallization using ethanol (Compound **1**).

To a solution of compound **1** (10 mmol) in the presence of acetic acid (5 mL) was added 2-[(1H-1,3-benzodiazol-2-yl)amino]acetohydrazide (2) (10 mmol) which was previously synthesized by our group [26]. The reaction mixture was stirred at room temperature for 4 h. Following the completion of the reaction, the solid product was thoroughly washed with water and filtered. The crude compound was purified with recrystallization using ethanol (MVB1)

Yield: 91%. FT-IR (υmax, cm^−1^): 3221.85 (NH), 3169. 81 (NH), 3070.59 (ar-CH), 1690.45 (C=O), 1653.61 (C=O), 1633.77, 1614.21, 1601.42 (C=N). 1H NMR (DMSO-d6, δppm): 3.70 (s, 2H, CH2), 6.72–6.76 (m, 3H, arH), 6.88 (t, 3H, J = 8.0 Hz, CH + arH), 7.14–7.19 (m, 4H, CH + arH), 7.33 (d, 1H, J = 4.0 Hz, arH), 7.63 (d, 1H, J = 4.0 Hz, arH), 7.76 (d, 1H, J = 8.0 Hz, arH), 8.05 (s, 1H, CH), 8.56 (s, 1H, OH), 9.26 (s, 1H, NH), 9.71 (s, 1H, NH), 11.23 (s, 1H, NH). 13C NMR (DMSO-d6, δppm): 42.51, 111.26, 114.16, 116.34, 119.61,121.22, 122.07, 122.52, 123.28, 124.61, 126.53, 128.11, 128.92, 130.65, 133.60, 133.82, 136.33, 136,64, 137.90, 148,87, 153.18, 155.50, 156.99, 158.44, 162.62, 168.34. MALDI-TOF-MS: 580.93759 ([M + Na]^+^)

#### 4.2.2. N′-[(E)-(2-Hydroxyphenyl)methylidene]-2-(4-Phenylpiperazin-1-yl)acetohydrazid (MVB2)

To a solution of 2-(4-phenylpiperazin-1-yl)acetohydrazide (**3**) (10 mmol) [27] in ethanol was added 2-hydroxy benzaldehyde (10 mmol) and 3–4 drops of glacial acetic acid as a catalyst. This was then stirred under reflux for 10 h (the reaction was followed by TLC using CHCl3:EtOH (5:1)). After removing the solvent under reduced pressure, the obtained solid was purified by recrystallization from methanol.

Yield: 82%. FT-IR (υmax, cm^−1^): 3220.30 (NH), 3022.37 (ar-CH), 1675.96 (C=O), 1612.78 (C=N). 1H NMR (DMSO-d6, δppm): 2.60–2.70 (m, 4H, 2CH2), 2.99–3.06 (m, 4H, 2CH2), 3.12 (s, 2H, CH2), 6.79 (dd, 2H, J1 = 2.0 Hz, J2 = 2.0 Hz, arH), 6.86 (dd, 2H, J1 = 3.2 Hz, J2 = 2.8 Hz, arH), 7.60 (d, 1H, J = 2.0 Hz, arH), 7.62 (s, 2H, arH), 7.71 (d, 1H, J = 8.4 Hz, arH), 7.81 (d, 1H, J = 8.0 Hz, arH), 8.68 (s, 1H, CH), 9.67 (s, 1H, OH), 11.02 (s, 1H, NH). 13C NMR (DMSO-d6, δppm): 48.44, 53.25, 60.76, 115.82, 116.80, 119.01, 119.24, 120.04, 129.36, 129.93, 131.73, 148.30, 151.42, 159.07, 165.99. MALDI-TOF-MS: 175.074504, 339.162926 ([M + 1]^+^), 361.12408.

### 4.3. Molecular Docking Studies

By analyzing each protein, those with appropriate solubility and clear inhibition/catalytic domains were identified. In the protein database (PDB), mTOR pathway PI3K, mTOR and AKT proteins were screened. Inhibitor and protein structures were revealed. Proteins were selected for subsequent analysis by calculation of binding energies in in silico calculations and molecular docking studies with known inhibitors or substrates. A grid box was then determined around the important amino acids in the catalytic site or inhibition suite and virtual inhibitor screening was performed. To determine the possible interactions of PI3K, AKT1, rapamycin and similar molecules targeting the mTOR pathway, molecular docking studies were performed using the open source Autodock Vina, as has been done many times before [56,57]. The 3D structure of PI3K (PDB:1E7V), AKT1 (PDB:3D0E) and mTOR (PDB:4DRI) proteins was downloaded from PDB in pdb format. It was opened in the Autodock Tools package and converted to pdbqt format. The water moved the molecules away. Then, scanning boxes around the catalytic site and/or inhibition package were determined and a script containing the vina conformation was prepared. Boxes of size 26Aox26Aox28Ao for AKT1, 32Aox30Aox30Ao for PI3K, and 26Aox26Aox26Ao for mTOR were designed around the co-crystallized inhibitor and substrate. Three-dimensional SDF files for rapamycin, 9 different known mTOR pathway inhibitors, and MVB1 and MVB2 compounds were prepared using Cactus. Molecular docking was automated using Autodock Vina and PaDelAdv.

### 4.4. In Silico ADME and Toxicity Analysis

ADME profiling and in silico toxicity studies were carried out through SwissADME [40], SwissTargetPrediction [58], ProTox-II [43] online web tools using the smiley codes of MVB1, MVB2 and rapamycin.

### 4.5. MTT Cell Proliferation Analysis

#### 4.5.1. 2D Culture Cell Proliferation Analysis

The Ishikawa cell line (Sigma Aldrich, 99040201-1VL, St. Louis, MO, USA) was cultured using Minimum Essential Media (MEM) (Gibco, 11095080, Miami, FL, USA) supplemented with 2 mM L-Glutamine (Sigma, 7513), 1% Non-Essential Amino Acids (NEAA) (Sigma, M7145), 5% Fetal Bovine Serum (FBS) (Sigma, F4135), and 1% antibiotic-antimycotic solution (Wisent Bioproducts, 450-115-EL, Saint-Jean-Baptiste, QC, Canada). The counted cells were seeded in 96-well plates at a density of 0.5 × 10^5^ cells per well. They were then incubated at 37 °C with 5% CO_2_ for 24 h to allow adherence to the plate and to achieve their normal morphology. All treatment doses were prepared by dissolving in Dimethyl sulfoxide (DMSO) (Sigma, D8418). Doses of MVB1, MVB2, and rapamycin (Medchem, HY-10219) at 100 µM, 50 µM, 40 µM, 30 µM, 20 µM, 10 µM, and 5 µM were added to the cells, along with 200 µL of medium, and incubated for another 24 h. Cells treated with a 0.1% Triton X solution (Sigma, T8787) served as the positive control group, while cells exposed only to the medium were designated as the negative control group. At the end of the incubation period, the MTT protocol was applied as in our previous study [59,60,61]. Inhibitory concentration (IC_50_), statistics and graphs of the compounds and rapamycin that caused a 50% decrease in the proliferation of cells were recorded using the GraphPad Prism 9.0.1 program.

#### 4.5.2. 3D Culture Cell Proliferation Analysis

The non-adhesive surface technique was used to create 3D tumor-like models, also called spheroids [62]. A total of 5 × 10^3^ cells per well were seeded in 200 uL medium in an agarose-coated 96-well plate, then cultured in an incubator at 37 °C and 5% CO_2_. Following the formation of spherical forms on the third day after seeding, the medium was replaced by half. On day 4, spheres of equal size, reaching 400 µm in diameter, were selected and transferred to the wells of another 96-well sterile cell culture plate. Diameter measurements were made with Zeiss Axiovert 5. Doses of 200 μM, 100 μM, 50 μM, 40 μM, 30 μM, 20 μM MVB1, MVB2, and rapamycin were added to the spheroids along with 300 μL of medium and incubated for 24 h. At the end of the 24th hour, the plate was centrifuged at 100 rpm for 1 min to ensure that the spheroids settled to the bottom of the wells. After aspiration of the medium, compound mixtures, and rapamycin, the MTT protocol was applied as specified in Section 4.5.1, and IC_50_ values were determined. Additionally, the diameter measurements of all spheroids on the 4th day (before exposure to the compounds) and the 5th day (after exposure to the compounds) were measured and compared statistically.

### 4.6. Western Blot Analysis

#### 4.6.1. 2D Cell Culture Model

When the cells cultured in T25 flasks reached 80% saturation, they were incubated for 24 h with concentrations of IC_50_ values determined in 2D cultures of MVB1, MVB2 and rapamycin. At the end of the 24th hour, the cells collected from the cell scraper were centrifuged at 1500 rpm for 5 min and the supernatant was removed. Western blot protocol was applied to the resulting cell pellet as in our previous study [63]. mTOR Polyclonal Antibody (1:200, BT-AP05644), PI 3-kinase p85a Polyclonal Antibody (1:200, BT-AP07139) and Akt1 Polyclonal Antibody (1:200, BT-AP00334) were diluted in non-fat milk as primary antibodies. Protein amount control was performed using β-Actin (CST, mAb#4970). Anti-Rabbit HRP-linked secondary antibody (Sigma Aldrich, RABHRP1) was used as the secondary antibody. Blots were analyzed with the Chemidoc MP imaging system (1708280) (BioRad Life Sciences Research, Hercules, CA, USA). The recorded images were analyzed with the Image J software (US National Institutes of Health, Bethesda, MD, USA).

#### 4.6.2. 3D Cell Culture Model

The diameters of the spheroids cultured as specified in Section 4.5.2 were measured at the end of the 4th day. Spheroids of equal size, reaching a diameter of 400 µm, were selected and transferred to the wells of another sterile 96-well cell culture plate. Concentrations of IC_50_ values determined in spheroid cultures of MVB1, MVB2, and rapamycin were prepared in 200 µL of medium and added to the wells. Twenty-four hours of incubation were provided. After applying the western blot protocol to the collected spheroids, as specified in Section 4.6.1, the membranes were incubated with ECL Western imaging solution for 5 min and then imaged using automatic exposure with the Chemidoc MP imaging system. The recorded images were analyzed with the Image J 1.4.3.67 program.

### 4.7. Immunofluorescence Staining

#### 4.7.1. 2D Cell Culture Model

Covered glass slides with 12 wells (Ibidi, 81201, Grueling, Germany) were used to perform and visualize the stainings. Cells were cultivated in 200 µL medium, with 104 cells per well. The cells were incubated in the incubator for two days to gain their normal morphology and reach confluency. Concentrations of IC_50_ values determined in 2D cultures of MVB1, MVB2 compounds and rapamycin were prepared in 200 µL of medium and added to the cells. Incubation was provided for 24 h. At the end of the incubation period, the immunofluorescence staining protocol was applied as in our previous study [62]. mTOR Polyclonal Antibody (BT-AP05644), PI 3-kinase p85a Polyclonal Antibody (BT-AP07139) and Akt1 Polyclonal Antibody (BT-AP00334), diluted 1:200 in antibody dilution solution (Sigma, U3510), were used as primary antibodies. Goat Anti-Rabbit A488(ab150077) was used as secondary antibody and DAPI (Sigma, F6057) was used for nuclear staining. Imaging was performed through a confocal microscope (Zeiss LSM 800). The recorded images were analyzed with the Image J program.

#### 4.7.2. 3D Cell Culture Model

The diameters of the spheroids cultured until the 4th day were measured, and spheroids of equal size reaching 400 µm in diameter were selected and transferred to the wells of covered glass slides with 12 wells. Concentrations of IC50 values of MVB1, MVB2 compounds and rapamycin determined in spheroid cultures were prepared in 200 µL of medium and added to the spheroids. Incubation was provided for 24 h. At the end of the 24th hour, the compound-medium and rapamycin-medium solutions were aspirated. Then, the immunofluorescence staining protocol was applied as specified in Section 4.7.1. Thirty sections were taken from the spheroids using the digital sectioning method (z-stack). Imaging was performed through a confocal microscope (Zeiss LSM 800). The recorded images were analyzed with the Image J program.

## 5. Conclusions

Although compounds MVB1 and MVB2 had the same bioavailability score in ADME results, MVB1 was more effective at binding to pathway proteins by −10.5 kcal/mol and exhibited less metabolic toxicity in in silico toxicity prediction results. It also exhibited consistent results in 2D and 3D cultures in reducing PI3K protein levels. This may strengthen the potential of the MVB1 compound as a new PI3K inhibitor, if supported by future in vivo experiments.

## Figures and Tables

**Table 1:** Numerical values of the compounds’ docking to proteins.

Compounds	PI3K (PDB:1E7V)	AKT (PDB:3D0E)	mTOR (PDB:4DRI)	Average (kcal/mol)
Rapamycin	−6.5	−7.05	−9.55	−7.7
MVB1	−9.8	−10.2	−11.6	−10.5
MVB2	−7.7	−8.1	−9.6	−8.5
Copanlisib	−8.3	−8.6	−9.8	−8.9
Cromolyn	−8.3	−8.9	−9.9	−9.0
GSK690693	−8.7	−7.0	−9.3	−8.3
Myricetin	−7.9	−8.3	−8.3	−8.2
Naproxen	−8.1	−7.7	−8.2	−8.0
Perifosine	−5.7	−5.5	−6.7	−6.0
PF−04802367	−7.2	−6.6	−8.1	−7.3
Quercetin	−8.3	−8.7	−8.9	−8.6
Wortmannin	−8.3	−7.6	−9.7	−8.5

**Table 2:** Toxicity classification results of MVB1, MVB2 and Rapamycin.

Compounds	Predicted LD50 (mg/kg)	Predicted Toxicity Class	Average Similarity (%)	Prediction Accuracy (%)
MVB1	3009	5	35.29	23
MVB2	1400	4	56.45	67.38
Rapamycin	2500	5	100	100

**Table 3:** Antiproliferative activity results of MVB1, MVB2 and rapamycin against Ishikawa cell line.

2D Cell Culture	IC_50_ (µM)/SEM ^a^	LogIC_50_ (µM)/SEM ^a^
MVB1	8.3/±0.5	0.9/±0.03
MVB2	9.0/±1.2	0.9/±0.06
Rapamycin	21.5/±0.6	1.3/±0.01
3D Cell Culture		
MVB1	49.9/±2.8	1.6/±0.02
MVB2	20.6/±1.9	1.3/±0.04
Rapamycin	38.5/±2.0	1.5/±0.02

^a^: Standard Error Mean.

**Table 4:** Percentage of cell viability for all doses of MVB1, MVB2, and rapamycin compounds in 2D culture.

Cell Viability (%)/SEM ^a^ Compound	5 µM	10 µM	20 µM	30 µM	40 µM	50 µM	100 µM
MVB1	61.5/±0.4	37.8/±4.2	27.1/±2	23.3/±0.6	19/±0.9	18.4/±0	13.5/±1.2
MVB2	59.4/±0.9	36.4/±0.9	29.5/±1.1	27.8/±1.2	27.8/±3.4	22.7/±2.5	27.6/±5.7
Rapamycin	99.6/±1.1	79.3/±3.1	61.4/±1.6	29.8/±0.9	12.5/±1.4	10.3/±1.7	9.5/±0.2

^a^: Standard Error Mean.

**Table 5:** Percentage of cell viability for all doses of MVB1, MVB2, and rapamycin compounds in 3D culture.

Cell Viability (%)/SEM ^a^ Compound	20 µM	30 µM	40 µM	50 µM	100 µM	200 µM
MVB1	63.3/±0.8	56.5/±1.3	54.1/±0.1	49.9/±1.1	45.3/±0	25.7/±0.2
MVB2	57.2/±0.5	37.8/±0.6	26.7/±0.2	25.6/±1	23.3/±0.7	18.9/±0.7
	**10 µM**	**20 µM**	**30 µM**	**40 µM**	**50 µM**	**100 µM**
Rapamycin	83.6/±1	61.3/±0.4	50.3/±0.8	44.9/±0.7	43.3/±0.1	38.4/±0.7

^a^: Standard Error Mean.

## Data Availability

Dataset available on request from the authors.

## Supplementary Materials

The following supporting information can be downloaded at: https://www.mdpi.com/article/10.3390/ph17121562/s1. Figure S1: FTIR spectra of MBV-1, 1H NMR spectra of MVB-1, 13C NMR spectra of MVB-1, MS spectra of MVB-1, FTIR spectra of MBV-2, 1H NMR spectra of MVB-2, 13C NMR spectra of MVB-2, MS spectra of MVB-2. Figure S2: ADME parameters of the compounds MVB1. Figure S3: ADME parameters of the compounds MVB2. Figure S4: ADME parameters of the Rapamycin. Figure S5: Toxicity prediction results of MVB1. Figure S6: Toxicity prediction results of MVB2. Figure S7: Toxicity prediction results of rapamycin.
