# Supplementary material for: mTOR Pathway Inhibition, Anticancer Activity and In Silico Calculations of Novel Hydrazone Derivatives in Two- and Three-Dimensional Cultured Type 1 Endometrial Cancer Cells
Source: Pharmaceuticals (Basel). 2024 Nov 21;17(12):1562. doi: 10.3390/ph17121562 (PMC11678851; doi:10.3390/ph17121562)
Supplement: Supplementary file 1 [file pharmaceuticals-17-01562-s001.zip › pharmaceuticals-3250262-supplementary.pdf]

(A)

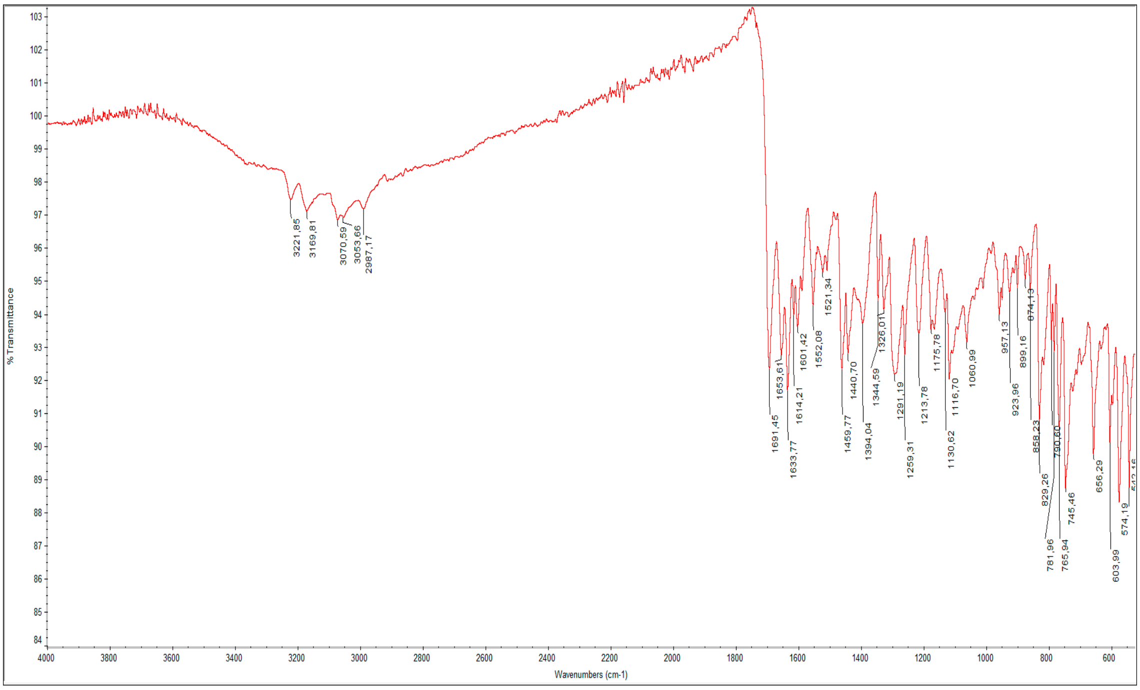
% Transmittance
Wavenumbers (cm-1)
3221,85
3169,81
3070,59
3053,66
2987,17
1691,45
1653,61
1633,77
1614,21
1601,42
1552,08
1521,34
1459,77
1440,70
1394,04
1344,59
1326,01
1291,19
1259,31
1213,78
1175,78
1130,62
1116,70
1060,99
957,13
923,96
899,16
874,13
858,23
829,26
790,60
781,96
765,94
745,46
656,29
603,99
574,19

(B)

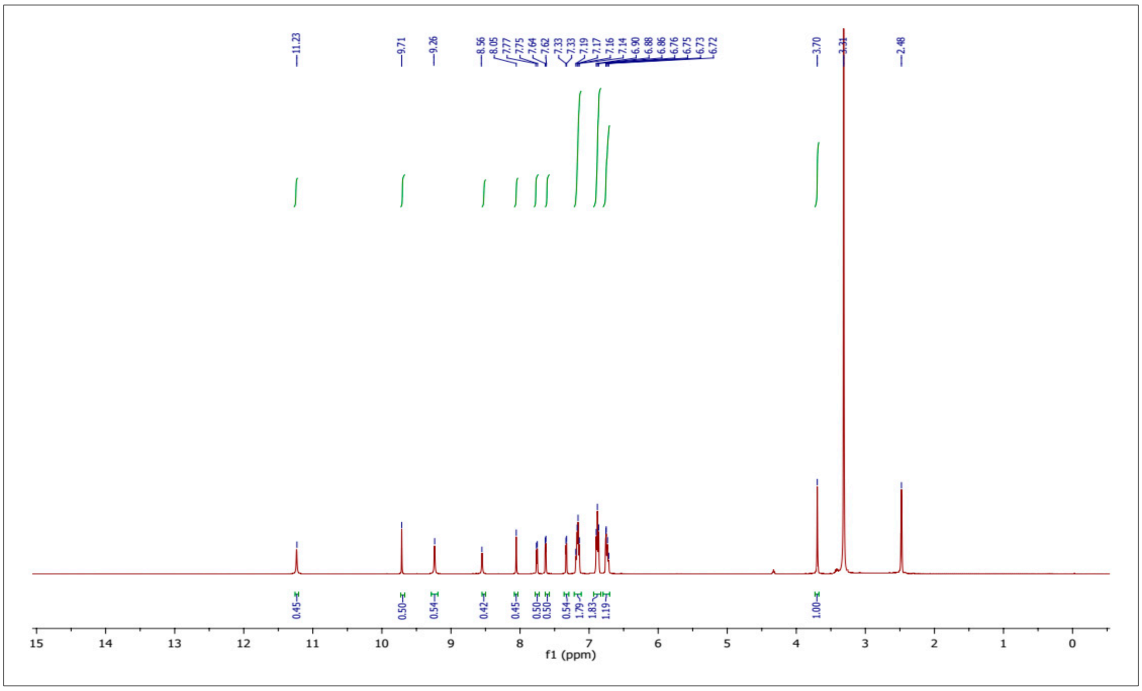
11.23
9.71
9.26
8.56
8.05
7.77
7.75
7.64
7.62
7.33
7.33
7.19
7.17
7.16
7.14
6.90
6.88
6.86
6.76
6.75
6.73
6.72
3.70
3.31
2.48
0.45
0.50
0.54
0.42
0.45
0.50
0.50
0.54
1.79
1.83
1.19
1.00
f1 (ppm)

(C)

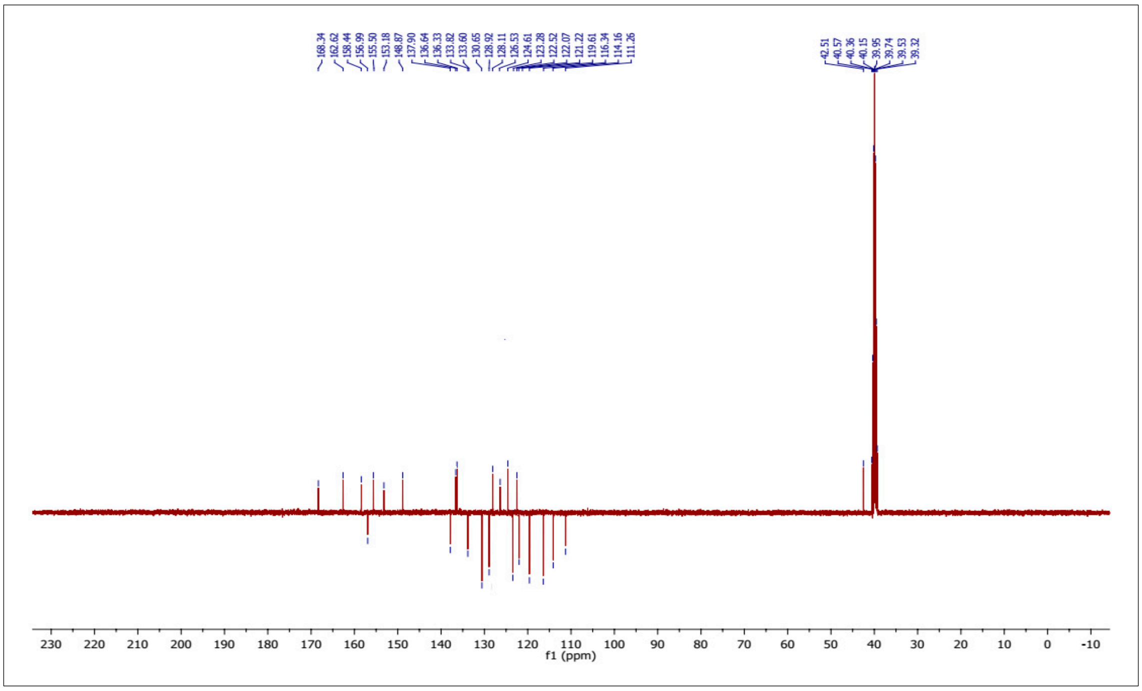
168.34
162.62
158.44
156.99
155.50
153.18
148.87
137.90
136.64
136.33
133.82
133.60
130.65
128.92
128.11
126.53
124.61
123.28
122.52
122.07
121.22
119.61
116.34
114.16
111.26
42.51
40.57
40.36
40.15
39.95
39.74
39.53
39.32
230 220 210 200 190 180 170 160 150 140 130 120 110 100 90 80 70 60 50 40 30 20 10 0 -10
f1 (ppm)

(D)

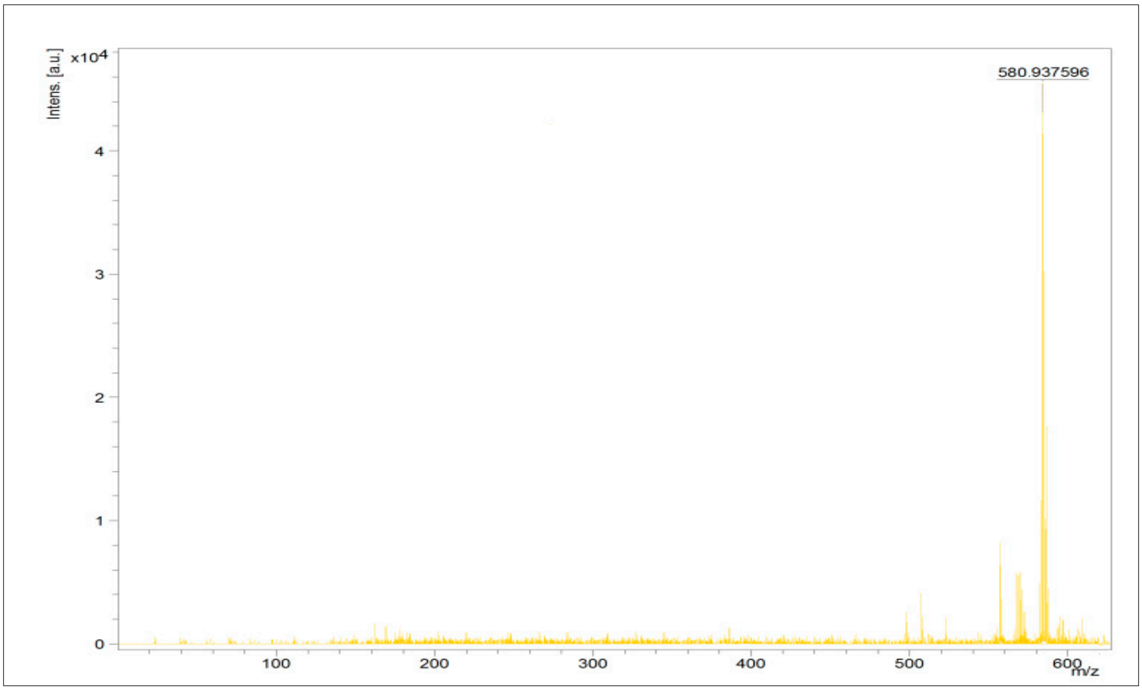
Intens. [a.u.]
x10^4
580.937596
0 1 2 3 4
100 200 300 400 500 600
m/z

(E)

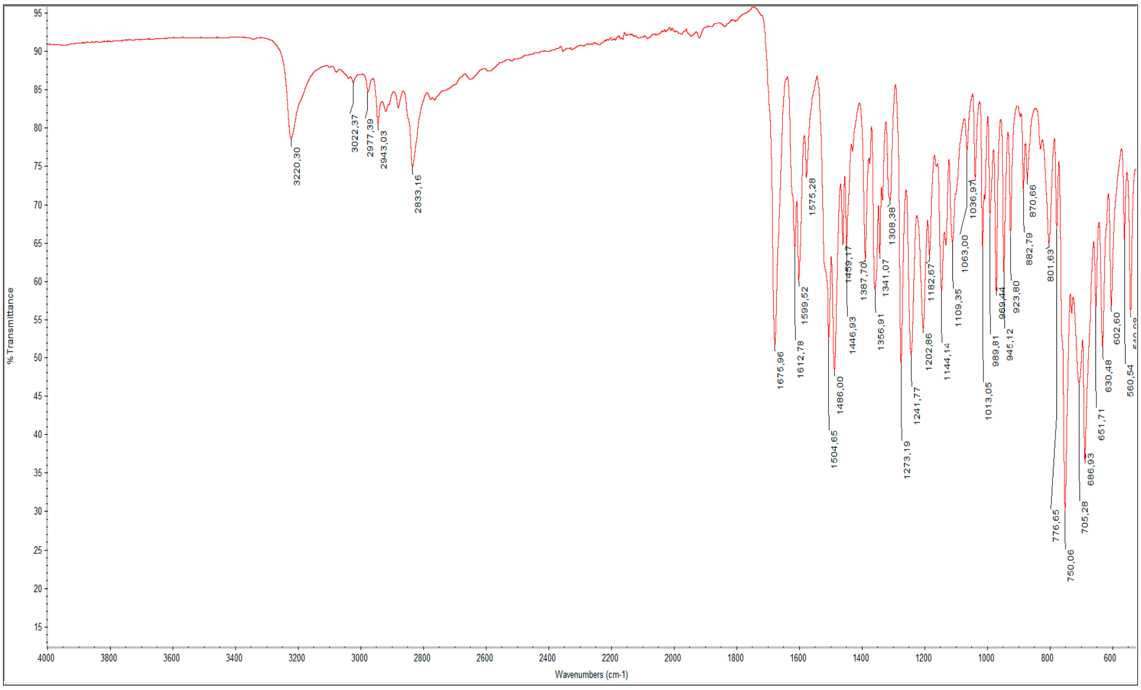
% Transmittance
Wavenumbers (cm-1)
3220,30
3022,37
2977,39
2943,03
2833,16
1675,96
1612,78
1599,52
1575,28
1504,65
1486,00
1459,17
1446,93
1387,70
1356,91
1341,07
1308,38
1273,19
1241,77
1202,86
1182,67
1144,14
1109,35
1063,00
1036,97
1013,05
989,81
969,44
945,12
923,80
882,79
870,66
801,63
776,65
750,06
705,28
686,93
651,71
630,48
602,60
560,54

(F)

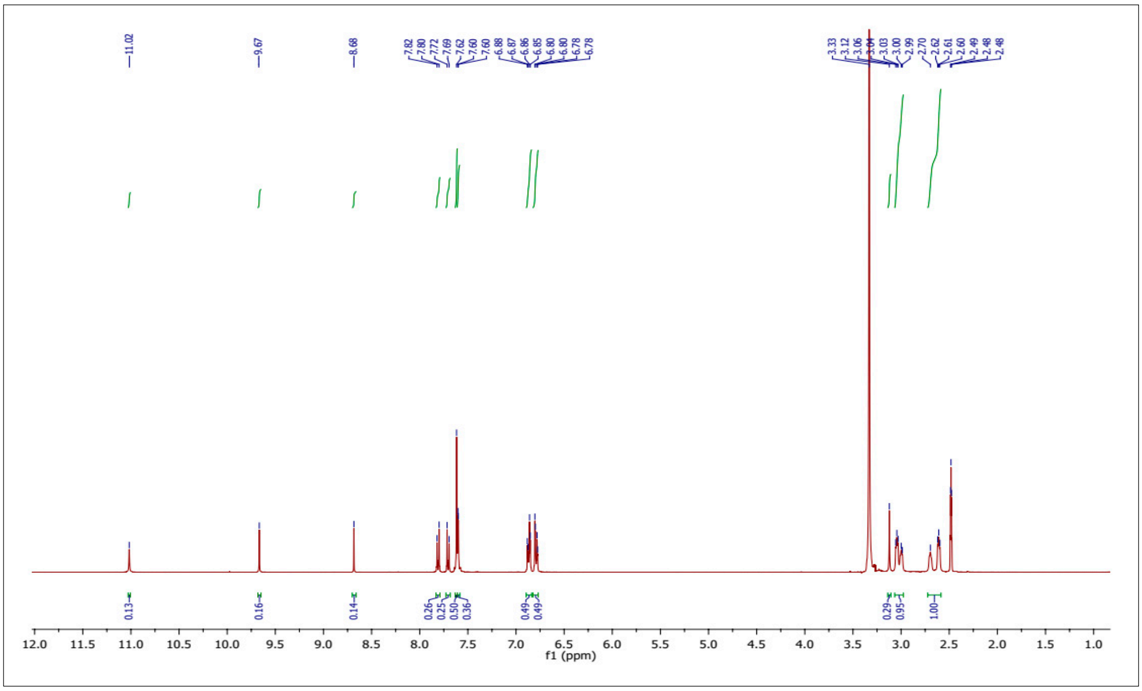
11.02
9.67
8.68
f1 (ppm)

(G)

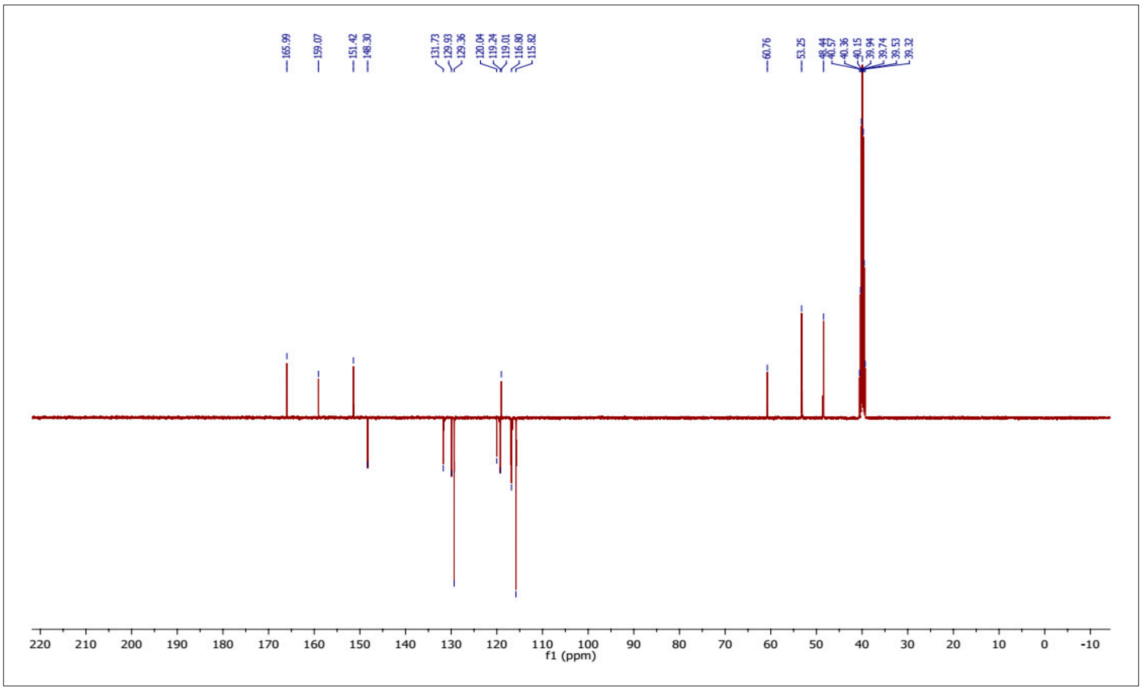

(H)

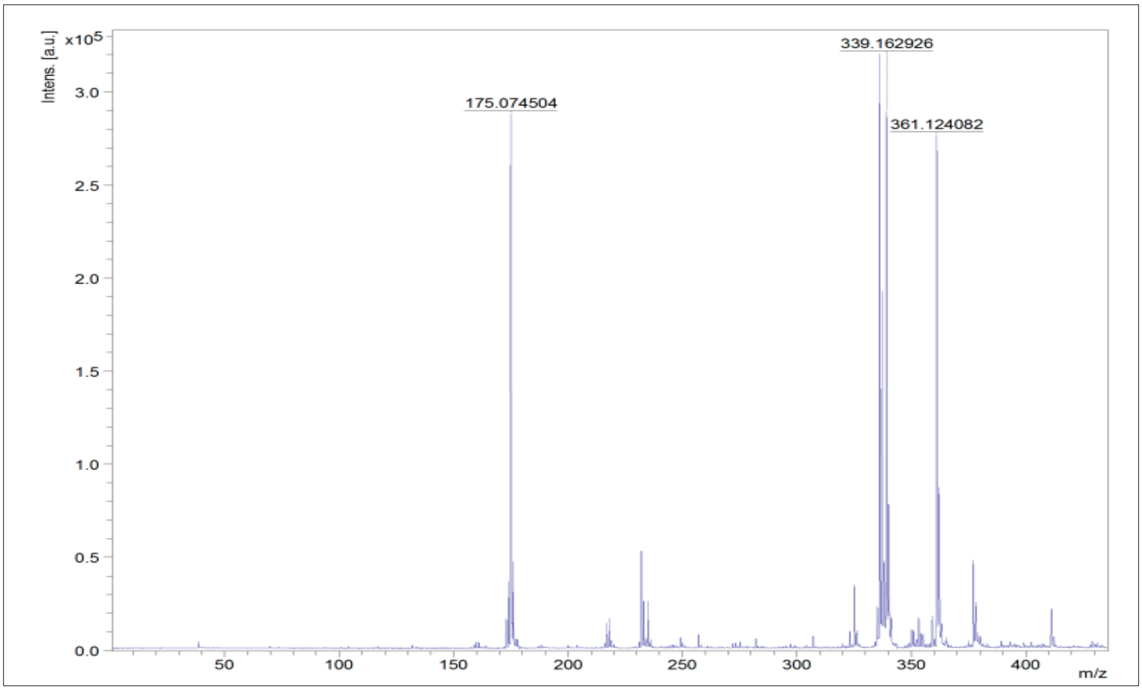

Supplementary Figure S1. (A) FTIR spectra of MBV-1. (B) ^{1}H NMR spectra of MVB-1. (C) ^{13}C NMR spectra of MVB-1. (D) MS spectra of MVB-1. (E) FTIR spectra of MBV-2. (F) ^{1}H NMR spectra of MVB-2. (G) ^{13}C NMR spectra of MVB-2. (H) MS spectra of MVB-2.

Molecule 1

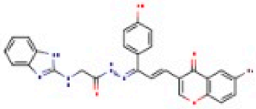

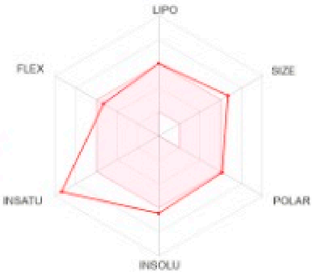

SMILES O=C(NN=C(c1ccc(cc1)O)/C=C/c1coc2c(c1=O)cc(cc2)Br)CNc1nc2c([nH]1)cccc2

Physicochemical Properties	
Formula	C27H20BrN5O4
Molecular weight	558.38 g/mol
Num. heavy atoms	37
Num. arom. heavy atoms	25
Fraction Csp3	0.04
Num. rotatable bonds	8
Num. H-bond acceptors	6
Num. H-bond donors	4
Molar Refractivity	146.37
TPSA	132.61 Å²

Lipophilicity	
Log $P_{o/w}$ (iLOGP)	3.11
Log $P_{o/w}$ (XLOGP3)	5.06
Log $P_{o/w}$ (WLOGP)	4.48
Log $P_{o/w}$ (MLOGP)	2.32
Log $P_{o/w}$ (SILICOS-IT)	5.15
Consensus Log $P_{o/w}$	4.03

Water Solubility	
Log S (ESOL)	-6.46
Solubility	1.93e-04 mg/ml ; 3.45e-07 mol/l
Class	Poorly soluble
Log S (Ali)	-7.59
Solubility	1.45e-05 mg/ml ; 2.59e-08 mol/l
Class	Poorly soluble
Log S (SILICOS-IT)	-9.83
Solubility	8.30e-08 mg/ml ; 1.49e-10 mol/l
Class	Poorly soluble

Pharmacokinetics	
GI absorption	Low
BBB permeant	No
P-gp substrate	No
CYP1A2 inhibitor	No
CYP2C19 inhibitor	Yes
CYP2C9 inhibitor	Yes
CYP2D6 inhibitor	No
CYP3A4 inhibitor	No
Log K_p (skin permeation)	-6.11 cm/s

Druglikeness	
Lipinski	Yes; 1 violation: MW>500
Ghose	No; 2 violations: MW>480, MR>130
Veber	Yes
Egan	No; 1 violation: TPSA>131.6
Muegge	No; 1 violation: XLOGP3>5
Bioavailability Score	0.55

Medicinal Chemistry	
PAINS	1 alert: hzone_phenol_B
Brenk	1 alert: imine_1
Leadlikeness	No; 3 violations: MW>350, Rotors>7, XLOGP3>3.5
Synthetic accessibility	4.07

Supplementary Figure S2. ADME parameters of the compounds MVB1.

Molecule 1

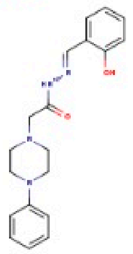

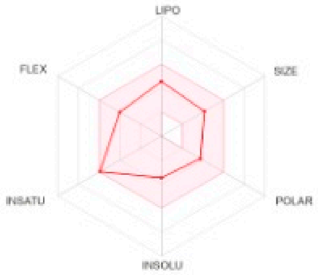

SMILES O=C(CN1CCN(CC1)c1ccccc1)NN=Cc1ccccc1O

Physicochemical Properties	
Formula	C19H22N4O2
Molecular weight	338.40 g/mol
Num. heavy atoms	25
Num. arom. heavy atoms	12
Fraction Csp3	0.26
Num. rotatable bonds	6
Num. H-bond acceptors	4
Num. H-bond donors	2
Molar Refractivity	105.51
TPSA	68.17 Å²

Lipophilicity	
Log $P_{o/w}$ (iLOGP)	2.62
Log $P_{o/w}$ (XLOGP3)	2.48
Log $P_{o/w}$ (WLOGP)	0.90
Log $P_{o/w}$ (MLOGP)	1.46
Log $P_{o/w}$ (SILICOS-IT)	1.92
Consensus Log $P_{o/w}$	1.88

Water Solubility	
Log *S* (ESOL)	-3.46
Solubility	1.17e-01 mg/ml ; 3.47e-04 mol/l
Class	Soluble
Log *S* (Ali)	-3.56
Solubility	9.40e-02 mg/ml ; 2.78e-04 mol/l
Class	Soluble
Log *S* (SILICOS-IT)	-4.59
Solubility	8.74e-03 mg/ml ; 2.58e-05 mol/l
Class	Moderately soluble

Pharmacokinetics	
GI absorption	High
BBB permeant	No
P-gp substrate	Yes
CYP1A2 inhibitor	No
CYP2C19 inhibitor	No
CYP2C9 inhibitor	No
CYP2D6 inhibitor	Yes
CYP3A4 inhibitor	No
Log K_p (skin permeation)	-6.60 cm/s

Druglikeness	
Lipinski	Yes; 0 violation
Ghose	Yes
Veber	Yes
Egan	Yes
Muegge	Yes
Bioavailability Score	0.55

Medicinal Chemistry	
PAINS	1 alert: hzone_phenol_A
Brenk	1 alert: imine_1
Leadlikeness	Yes
Synthetic accessibility	2.93

Supplementary Figure S3. ADME parameters of the compounds MVB2.

Molecule 1

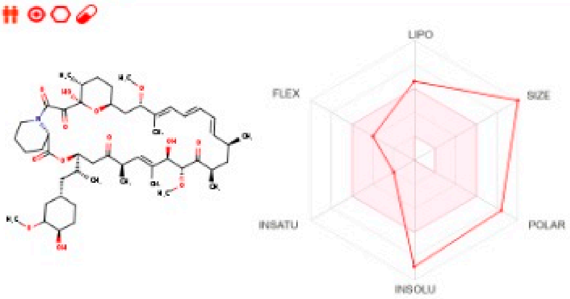

SMILES: COC1C[C@@H](CC[C@H]1O)C[C@H]([C@H]1OC(=O)[C@@H]2CCCCN2C(=O)C(=O)[C@]2(O)O[C@@H](CC[C@H]2C)C[C@H](OC)/C(=C/C=C/C=C/[C@H](C[C@H](C(=O)[C@@H]([C@@H](/C(=C/[C@H](C(=O)C1)C)/C)O)OC)C)C)/C)C

Physicochemical Properties	
Formula	C51H79NO13
Molecular weight	914.17 g/mol
Num. heavy atoms	65
Num. arom. heavy atoms	0
Fraction Csp3	0.75
Num. rotatable bonds	6
Num. H-bond acceptors	13
Num. H-bond donors	3
Molar Refractivity	253.68
TPSA	195.43 Å²

Lipophilicity	
Log $P_{o/w}$ (iLOGP)	6.33
Log $P_{o/w}$ (XLOGP3)	6.02
Log $P_{o/w}$ (WLOGP)	5.80
Log $P_{o/w}$ (MLOGP)	1.00
Log $P_{o/w}$ (SILICOS-IT)	3.66
Consensus Log $P_{o/w}$	4.56

Water Solubility	
Log *S* (ESOL)	-8.90
Solubility	1.14e-06 mg/ml ; 1.25e-09 mol/l
Class	Poorly soluble
Log *S* (Ali)	-9.90
Solubility	1.15e-07 mg/ml ; 1.25e-10 mol/l
Class	Poorly soluble
Log *S* (SILICOS-IT)	-5.58
Solubility	2.38e-03 mg/ml ; 2.61e-06 mol/l
Class	Moderately soluble

Pharmacokinetics	
GI absorption	Low
BBB permeant	No
P-gp substrate	Yes
CYP1A2 inhibitor	No
CYP2C19 inhibitor	No
CYP2C9 inhibitor	No
CYP2D6 inhibitor	No
CYP3A4 inhibitor	No
Log K_p (skin permeation)	-7.60 cm/s

Druglikeness	
Lipinski	No; 2 violations: MW>500, NorO>10
Ghose	No; 4 violations: MW>480, WLOGP>5.6, MR>130, #atoms>70
Veber	No; 1 violation: TPSA>140
Egan	No; 1 violation: TPSA>131.6
Muegge	No; 4 violations: MW>600, XLOGP3>5, TPSA>150, H-acc>10
Bioavailability Score	0.17

Medicinal Chemistry	
PAINS	0 alert
Brenk	2 alerts: diketo_group, isolated_alkene
Leadlikeness	No; 2 violations: MW>350, XLOGP3>3.5
Synthetic accessibility	10.00

Supplementary Figure S4. ADME parameters of the rapamycin.

ProTox-3.0 - Prediction of TOXicity of chemicals

Classification	Target	Shorthand	Prediction	Probability
Organ toxicity	Hepatotoxicity	dili	Active	0.51
Organ toxicity	Neurotoxicity	neuro	Active	0.68
Organ toxicity	Nephrotoxicity	nephro	Active	0.57
Organ toxicity	Respiratory toxicity	respi	Active	0.79
Organ toxicity	Cardiotoxicity	cardio	Inactive	0.71
Toxicity end points	Carcinogenicity	carcino	Active	0.50
Toxicity end points	Immunotoxicity	immuno	Active	0.60
Toxicity end points	Mutagenicity	mutagen	Inactive	0.56
Toxicity end points	Cytotoxicity	cyto	Inactive	0.58
Toxicity end points	BBB-barrier	bbb	Active	0.57
Toxicity end points	Ecotoxicity	eco	Inactive	0.67
Toxicity end points	Clinical toxicity	clinical	Active	0.64
Toxicity end points	Nutritional toxicity	nutri	Inactive	0.57
Tox21-Nuclear receptor signalling pathways	Aryl hydrocarbon Receptor (AhR)	nr_ahr	Inactive	0.81
Tox21-Nuclear receptor signalling pathways	Androgen Receptor (AR)	nr_ar	Inactive	0.96
Tox21-Nuclear receptor signalling pathways	Androgen Receptor Ligand Binding Domain (AR-LBD)	nr_ar_lbd	Inactive	0.98
Tox21-Nuclear receptor signalling pathways	Aromatase	nr_aromatase	Inactive	0.93
Tox21-Nuclear receptor signalling pathways	Estrogen Receptor Alpha (ER)	nr_er	Inactive	0.81
Tox21-Nuclear receptor signalling pathways	Estrogen Receptor Ligand Binding Domain (ER-LBD)	nr_er_lbd	Inactive	0.94
Tox21-Nuclear receptor signalling pathways	Peroxisome Proliferator Activated Receptor Gamma (PPAR-Gamma)	nr_ppar_gamma	Inactive	0.93
Tox21-Stress response pathways	Nuclear factor (erythroid-derived 2)-like 2/ antioxidant responsive element (nrf2/ARE)	sr_are	Inactive	0.92
Tox21-Stress response pathways	Heat shock factor response element (HSE)	sr_hse	Inactive	0.92
Tox21-Stress response pathways	Mitochondrial Membrane Potential (MMP)	sr_mmp	Inactive	0.83
Tox21-Stress response pathways	Phosphoprotein (Tumor Supressor) p53	sr_p53	Inactive	0.84
Tox21-Stress response pathways	ATPase family AAA domain-containing protein 5 (ATAD5)	sr_atad5	Inactive	0.89
Molecular Initiating Events	Thyroid hormone receptor alpha (THRα)	mie_thr_alpha	Inactive	0.90
Molecular Initiating Events	Thyroid hormone receptor beta (THRβ)	mie_thr_beta	Inactive	0.78
Molecular Initiating Events	Transtyretrin (TTR)	mie_ttr	Inactive	0.97
Molecular Initiating Events	Ryanodine receptor (RYR)	mie_ryr	Inactive	0.98
Molecular Initiating Events	GABA receptor (GABAR)	mie_gabar	Inactive	0.96
Molecular Initiating Events	Glutamate N-methyl-D-aspartate receptor (NMDAR)	mie_nmdar	Inactive	0.92
Molecular Initiating Events	alpha-amino-3-hydroxy-5-methyl-4-isoxazolepropionate receptor (AMPAR)	mie_ampar	Inactive	0.97
Molecular Initiating Events	Kainate receptor (KAR)	mie_kar	Inactive	0.99
Molecular Initiating Events	Achetylcholinesterase (AChE)	mie_ache	Active	0.51
Molecular Initiating Events	Constitutive androstane receptor (CAR)	mie_car	Inactive	0.98
Molecular Initiating Events	Pregnane X receptor (PXR)	mie_pxr	Inactive	0.92
Molecular Initiating Events	NADH-quinone oxidoreductase (NADHOX)	mie_nadhox	Inactive	0.97
Molecular Initiating Events	Voltage gated sodium channel (VGSC)	mie_vgsc	Inactive	0.95
Molecular Initiating Events	Na+/I- symporter (NIS)	mie_nis	Inactive	0.98
Metabolism	Cytochrome CYP1A2	CYP1A2	Inactive	0.85
Metabolism	Cytochrome CYP2C19	CYP2C19	Inactive	0.84
Metabolism	Cytochrome CYP2C9	CYP2C9	Inactive	0.67
Metabolism	Cytochrome CYP2D6	CYP2D6	Inactive	0.75
Metabolism	Cytochrome CYP3A4	CYP3A4	Inactive	0.80
Metabolism	Cytochrome CYP2E1	CYP2E1	Inactive	0.99

Supplementary Figure S5. Toxicity prediction results of MVB1.

ProTox-3.0 - Prediction of TOXicity of chemicals

Classification	Target	Shorthand	Prediction	Probability
Organ toxicity	Hepatotoxicity	dili	Inactive	0.65
Organ toxicity	Neurotoxicity	neuro	Active	0.77
Organ toxicity	Nephrotoxicity	nephro	Active	0.54
Organ toxicity	Respiratory toxicity	respi	Active	0.82
Organ toxicity	Cardiotoxicity	cardio	Inactive	0.71
Toxicity end points	Carcinogenicity	carcino	Active	0.57
Toxicity end points	Immunotoxicity	immuno	Inactive	0.65
Toxicity end points	Mutagenicity	mutagen	Inactive	0.56
Toxicity end points	Cytotoxicity	cyto	Inactive	0.58
Toxicity end points	BBB-barrier	bbb	Active	0.60
Toxicity end points	Ecotoxicity	eco	Inactive	0.57
Toxicity end points	Clinical toxicity	clinical	Active	0.7
Toxicity end points	Nutritional toxicity	nutri	Inactive	0.59
Tox21-Nuclear receptor signalling pathways	Aryl hydrocarbon Receptor (AhR)	nr_ahr	Inactive	0.82
Tox21-Nuclear receptor signalling pathways	Androgen Receptor (AR)	nr_ar	Inactive	0.95
Tox21-Nuclear receptor signalling pathways	Androgen Receptor Ligand Binding Domain (AR-LBD)	nr_ar_lbd	Inactive	0.99
Tox21-Nuclear receptor signalling pathways	Aromatase	nr_aromatase	Inactive	0.91
Tox21-Nuclear receptor signalling pathways	Estrogen Receptor Alpha (ER)	nr_er	Inactive	0.88
Tox21-Nuclear receptor signalling pathways	Estrogen Receptor Ligand Binding Domain (ER-LBD)	nr_er_lbd	Inactive	0.99
Tox21-Nuclear receptor signalling pathways	Peroxisome Proliferator Activated Receptor Gamma (PPAR-Gamma)	nr_ppar_gamma	Inactive	0.97
Tox21-Stress response pathways	Nuclear factor (erythroid-derived 2)-like 2/antioxidant responsive element (nrf2/ARE)	sr_are	Inactive	0.92
Tox21-Stress response pathways	Heat shock factor response element (HSE)	sr_hse	Inactive	0.92
Tox21-Stress response pathways	Mitochondrial Membrane Potential (MMP)	sr_mmp	Inactive	0.79
Tox21-Stress response pathways	Phosphoprotein (Tumor Supressor) p53	sr_p53	Inactive	0.91
Tox21-Stress response pathways	ATPase family AAA domain-containing protein 5 (ATAD5)	sr_atad5	Inactive	0.94
Molecular Initiating Events	Thyroid hormone receptor alpha (THRα)	mie_thr_alpha	Inactive	0.90
Molecular Initiating Events	Thyroid hormone receptor beta (THRβ)	mie_thr_beta	Inactive	0.78
Molecular Initiating Events	Transtyretrin (TTR)	mie_ttr	Inactive	0.97
Molecular Initiating Events	Ryanodine receptor (RYR)	mie_ryr	Inactive	0.98
Molecular Initiating Events	GABA receptor (GABAR)	mie_gabar	Inactive	0.96
Molecular Initiating Events	Glutamate N-methyl-D-aspartate receptor (NMDAR)	mie_nmdar	Inactive	0.92
Molecular Initiating Events	alpha-amino-3-hydroxy-5-methyl-4-isoxazolepropionate receptor (AMPAR)	mie_ampar	Inactive	0.97
Molecular Initiating Events	Kainate receptor (KAR)	mie_kar	Inactive	0.99
Molecular Initiating Events	Achetylcholinesterase (AChE)	mie_ache	Inactive	0.65
Molecular Initiating Events	Constitutive androstane receptor (CAR)	mie_car	Inactive	0.98
Molecular Initiating Events	Pregnane X receptor (PXR)	mie_pxr	Inactive	0.92
Molecular Initiating Events	NADH-quinone oxidoreductase (NADHOX)	mie_nadhox	Inactive	0.97
Molecular Initiating Events	Voltage gated sodium channel (VGSC)	mie_vgsc	Inactive	0.95
Molecular Initiating Events	Na+/I- symporter (NIS)	mie_nis	Inactive	0.98
Metabolism	Cytochrome CYP1A2	CYP1A2	Inactive	0.92
Metabolism	Cytochrome CYP2C19	CYP2C19	Inactive	0.79
Metabolism	Cytochrome CYP2C9	CYP2C9	Inactive	0.58
Metabolism	Cytochrome CYP2D6	CYP2D6	Active	0.64
Metabolism	Cytochrome CYP3A4	CYP3A4	Inactive	0.80
Metabolism	Cytochrome CYP2E1	CYP2E1	Inactive	0.99

Supplementary Figure S6. Toxicity prediction results of MVB2.

ProTox-3.0 - Prediction of TOXicity of chemicals

Classification	Target	Shorthand	Prediction	Probability
Organ toxicity	Hepatotoxicity	dili	Inactive	0.87
Organ toxicity	Neurotoxicity	neuro	Active	0.95
Organ toxicity	Nephrotoxicity	nephro	Active	0.75
Organ toxicity	Respiratory toxicity	respi	Active	0.98
Organ toxicity	Cardiotoxicity	cardio	Inactive	0.63
Toxicity end points	Carcinogenicity	carcino	Active	0.50
Toxicity end points	Immunotoxicity	immuno	Active	0.99
Toxicity end points	Mutagenicity	mutagen	Inactive	0.74
Toxicity end points	Cytotoxicity	cyto	Inactive	0.69
Toxicity end points	BBB-barrier	bbb	Inactive	0.93
Toxicity end points	Ecotoxicity	eco	Inactive	0.65
Toxicity end points	Clinical toxicity	clinical	Active	0.76
Toxicity end points	Nutritional toxicity	nutri	Active	0.70
Tox21-Nuclear receptor signalling pathways	Aryl hydrocarbon Receptor (AhR)	nr_ahr	Inactive	0.99
Tox21-Nuclear receptor signalling pathways	Androgen Receptor (AR)	nr_ar	Inactive	0.99
Tox21-Nuclear receptor signalling pathways	Androgen Receptor Ligand Binding Domain (AR-LBD)	nr_ar_lbd	Inactive	0.98
Tox21-Nuclear receptor signalling pathways	Aromatase	nr_aromatase	Active	1.0
Tox21-Nuclear receptor signalling pathways	Estrogen Receptor Alpha (ER)	nr_er	Inactive	0.73
Tox21-Nuclear receptor signalling pathways	Estrogen Receptor Ligand Binding Domain (ER-LBD)	nr_er_lbd	Inactive	0.91
Tox21-Nuclear receptor signalling pathways	Peroxisome Proliferator Activated Receptor Gamma (PPAR-Gamma)	nr_ppar_gamma	Inactive	0.95
Tox21-Stress response pathways	Nuclear factor (erythroid-derived 2)-like 2/ antioxidant responsive element (nrf2/ARE)	sr_are	Inactive	0.92
Tox21-Stress response pathways	Heat shock factor response element (HSE)	sr_hse	Inactive	0.92
Tox21-Stress response pathways	Mitochondrial Membrane Potential (MMP)	sr_mmp	Active	0.98
Tox21-Stress response pathways	Phosphoprotein (Tumor Supressor) p53	sr_p53	Inactive	0.89
Tox21-Stress response pathways	ATPase family AAA domain-containing protein 5 (ATAD5)	sr_atad5	Inactive	0.98
Molecular Initiating Events	Thyroid hormone receptor alpha (THRα)	mie_thr_alpha	Inactive	0.90
Molecular Initiating Events	Thyroid hormone receptor beta (THRβ)	mie_thr_beta	Inactive	0.78
Molecular Initiating Events	Transtyretrin (TTR)	mie_ttr	Inactive	0.97
Molecular Initiating Events	Ryanodine receptor (RYR)	mie_ryr	Inactive	0.98
Molecular Initiating Events	GABA receptor (GABAR)	mie_gabar	Inactive	0.96
Molecular Initiating Events	Glutamate N-methyl-D-aspartate receptor (NMDAR)	mie_nmdar	Inactive	0.92
Molecular Initiating Events	alpha-amino-3-hydroxy-5-methyl-4-isoxazolepropionate receptor (AMPAR)	mie_ampar	Inactive	0.97
Molecular Initiating Events	Kainate receptor (KAR)	mie_kar	Inactive	0.99
Molecular Initiating Events	Achetylcholinesterase (AChE)	mie_ache	Inactive	0.87
Molecular Initiating Events	Constitutive androstane receptor (CAR)	mie_car	Inactive	0.98
Molecular Initiating Events	Pregnane X receptor (PXR)	mie_pxr	Inactive	0.92
Molecular Initiating Events	NADH-quinone oxidoreductase (NADHOX)	mie_nadhox	Inactive	0.97
Molecular Initiating Events	Voltage gated sodium channel (VGSC)	mie_vgsc	Inactive	0.95
Molecular Initiating Events	Na+/I- symporter (NIS)	mie_nis	Inactive	0.98
Metabolism	Cytochrome CYP1A2	CYP1A2	Inactive	0.98
Metabolism	Cytochrome CYP2C19	CYP2C19	Inactive	0.91
Metabolism	Cytochrome CYP2C9	CYP2C9	Inactive	0.76
Metabolism	Cytochrome CYP2D6	CYP2D6	Inactive	0.83
Metabolism	Cytochrome CYP3A4	CYP3A4	Inactive	0.78
Metabolism	Cytochrome CYP2E1	CYP2E1	Inactive	0.99

Supplementary Figure S7. Toxicity prediction results of rapamycin.
